# Cross-sectional analysis of serum calcium levels for associations with left ventricular hypertrophy in normocalcemia individuals with type 2 diabetes

**DOI:** 10.1186/s12933-015-0200-9

**Published:** 2015-04-29

**Authors:** Junfeng Li, Nan Wu, Yintao Li, Kuanping Ye, Min He, Renming Hu

**Affiliations:** The Institute of Endocrinology and Diabetology, Huashan Hospital, Shanghai Medical College, Fudan University, 12 Middle Wulumuqi Road, Shanghai, 200040 China; Department of Medical Oncology, Shandong Cancer Hospital and Institute, Shandong Academy of Medical Sciences, Jinan, 250117 China

**Keywords:** Calcium, Left ventricular hypertrophy, Type 2 diabetes

## Abstract

**Background:**

Left ventricular hypertrophy (LVH) is prevalent in patients with type 2 diabetes mellitus (T2DM). Recent studies show that an increase in albumin-adjusted serum calcium level is associated with an elevated risk of T2DM. We speculate that increased serum calcium levels in T2DM patients are related to LVH prevalence.

**Methods:**

In this echocardiographic study, 833 normocalcemia and normophosphatemia patients with T2DM were enrolled. The associations between serum calcium and metabolic parameters, left ventricular mass index (LVMI), as well as the rate of LVH were examined using bivariate linear correlation, multivariate linear regression and logistic regression, respectively. The predictive performance of serum calcium for LVH was evaluated using the area under the receiver operating characteristic curve (AUC).

**Results:**

Patients with LVH have significantly higher serum calcium than those without LVH. Serum calcium was positively associated with total cholesterol, triglycerides, low-density lipoprotein cholesterol, serum uric acid, HOMA-IR and fasting plasma glucose. Multivariate linear regression analysis demonstrated that serum calcium was independently associated with LVMI (p < 0.001). In comparison with patients in the lowest serum calcium quartile, the odds ratio (OR) for LVH in patients in the highest quartile was 2.909 (95% CI 1.792-4.720; p < 0.001). When serum calcium was analyzed as a continuous variable, per 1 mg/dl increase, the OR (95% CI) for LVH was [2.400 (1.552-3.713); p < 0.001]. Serum calcium can predict LVH (AUC = 0.617; 95% CI (0.577-0.656); p < 0.001).

**Conclusions:**

Albumin-adjusted serum calcium is associated with an increased risk of LVH in patients with T2DM.

## Background

Left ventricular hypertrophy (LVH) is a threatening prognostic sign and an independent risk factor for cardiac death, coronary heart disease, ventricular dysrhythmias, and heart failure [1]. The presence of type 2 diabetes mellitus (T2DM) per se is independently associated with LVH, even in the absence of hypertension and obesity [2-4]. Several mechanisms such as insulin resistance, hyperlipidemia and diabetic kidney damage have been proposed to explain the relationship between T2DM and LVH. But after adjusting those potentially risk factors including glycaemia control, blood lipids, urinary albumin excretion rate and homeostasis model assessment insulin resistance (HOMA-IR), LVH is still a common condition in patients with T2DM [5,6]. Consequently, screening additional possible risk factors in the development of LVH in diabetic patients is an important subject of investigation.

Cumulative evidences reveal that an alteration in calcium homeostasis (indexed by serum calcium level) accompanies the diabetic state [7-10]. Serum calcium level is positively correlated with blood lipids and glucose levels in T2DM after adjustment for parathyroid hormone (PTH) as well as age, body weight, height, creatinine, albumin, or bone metabolism [8-10]. Moreover, an increase in serum calcium level is associated with an increased risk of T2DM independently of measured glucose [11,12], insulin secretion and insulin resistance [12].

Based on these findings, we speculate that an alteration in calcium homeostasis is associated with LVH prevalence, and we conduct a cross-sectional study to evaluate relations between serum calcium levels and LVH in T2DM patients.

## Methods

### Participants

A total of 833 subjects (486 men and 347 women) were included in this study. We recruited consecutive subjects who visited Huashan Hospital for education, evaluation, or treatment of T2DM from 2010 to 2014. To minimize the possibility that some abnormal conditions may influence the results, patients with any of the following conditions were excluded: 1) serum creatinine > 130 μmol/L (normal range: 50–130 μmol/L) or urine albumin per gram urine creatinine (Alb/Cr) > 300 mg/g; 2) a history of parathyroid disease or vitamin D-related disorders, as well as other diseases which can affect vitamin D absorption and metabolism; 3) medication history including vitamin D, bisphosphonate, estrogen replacement therapy and diuretics which may influence calcium metabolism within the past 1 month; 4) serum calcium out of normal range from central laboratory of Huashan hospital (8.42-10.42 mg/dl, or 2.10-2.60 mmol/l); 5) serum phosphate out of normal range from central laboratory of Huashan hospital (3.00-4.50 mg/dl, or 0.97-1.45 mmol/l); 6) a history of myocardial infarction, coronary artery bypass or angioplasty, atrial fibrillation, moderate and severe valvular heart disease, stroke or occlusive peripheral vascular disease, heart failure; 7) uncontrolled thyroid diseases, or active urinary tract infection. This study was approved by the ethical review board of Huashan Hospital and complied with the Helsinki declaration. Written informed consent was obtained from all participants.

### Biochemical measurements

A 12-hour overnight fasting venous blood sample was collected in all subjects. A morning fasting spot urine sample was collected once every month for three consecutive months to estimate the urinary albumin-to-urinary creatinine ratio. The creatinine, albumin, calcium, phosphate, uric acid, total cholesterol (TC), triglycerides (TG), low-density lipoprotein cholesterol (LDL-C), high-density lipoprotein cholesterol (HDL-C), and fasting plasma glucose (FPG) were measured by biochemical auto analyzer (Abbott C8000). The fasting insulin concentration was measured by electrochemiluminescence immunoassay technique (Roche Elecsys 2010). HbA1c was measured by high performance liquid chromatography (HPLC; Bio-Rad, Hercules, CA, USA). Serum calcium level was corrected according to the formula: albumin-adjusted serum calcium concentration (mg/dl) = measured serum calcium concentration (mg/dl) + 0.8 × [4 − serum albumin concentration (g/dl)] [11]. Insulin resistance was determined using the homeostatic model assessment: HOMA-IR = fasting plasma glucose (mmol/l) × fasting plasma insulin (mIU/L)/22.5 [13].

### Echocardiography

As recommended by the American Society of Echocardiography [14], with patients in partial left lateral decubitus positions, echocardiographic examination was performed under two-dimensional guided M-mode with a Vingmed System 5 Doppler echocardiographic unit (GE Vingmed Ultrasound, Horten, Norway). Left ventricular mass (LVM) was calculated by the Devereux formula [15]: LVM (g) = 0.8{1.04 [([LVIDD (left ventricular internal diameter, diastolic) + PWTD (posterior wall thickness, diastolic) + IVSD (inter ventricular septum, diastolic)]^3^ − LVIDD^3^)]} + 0.6. Relative wall thickness (RWT) was calculated as 2 × PWTD /LVIDD and increased RWT was defined as > 0.42 [16]. LVM index (LVMI) was derived by correcting LVM for body surface area [BSA (m^2^) = 0.007184 × Height (cm)^0.725^ × Weight (kg)^0.425^] [17]. LVH was defined as follows: LVMI > 115 g/m^2^ for men and LVMI > 95 g/m^2^ for women [16]. LV geometry was defined as “normal” (both RWT and LVMI normal), “concentric remodeling” (increased RWT but normal LVMI), “eccentric hypertrophy” (increased LVMI but normal RWT), and “concentric hypertrophy” (both LVMI and RWT increased) [16].

### Others

T2DM was defined as the onset of diabetes after the age of 35. Diabetes was diagnosed by the American Diabetes Association guideline [18]. Body mass index (BMI, kg/m^2^) was defined as weight divided by square height. Obesity was defined as BMI ≥ 28 kg/m^2^ according to Chinese standard [19]. Smoking was defined as “ever smoked” as compared to “never smoked”. Hypertension was defined as systolic blood pressure (SBP) ≥ 140 mmHg and/or diastolic blood pressure (DBP) ≥90 mmHg, or current antihypertensive therapy. Micro-albuminuria was defined as Alb/Cr between 30 and 300 mg/g, and macro-albuminuria was defined as Alb/Cr > 300 mg/g. Dyslipidemia was defined as HDL-C < 1.04 mmol/l, LDL-C ≥ 4.14 mmol/l, or TG ≥ 2.26 mmol/l [20].

### Data analysis

Descriptive statistics of mean and standard deviation (SD) were calculated for continuous variables, as well as frequencies and percentages for categorical variables. Kolmogorov-Smirnov Test was used to judge whether continuous variable followed normal distribution. HOMA-IR and Alb/Cr were logarithmically transformed to approximate normal distribution before analysis. Differences in continuous variables were determined by independent-samples *T*-test or One-way ANOVA. If data were non-normal distributed or not met the homogeneity of variances, nonparametric test was used. Chi-square test was used for categorical variables. The associations between serum calcium and metabolic parameters were examined using bivariate linear correlation (Pearson correlation). Using factors identified by the initial univariate linear regression, a stepwise multiple linear regression was carried out to determine independent variable’s contribution to LVMI. Collinearity diagnostics was used to confirm whether the predictors are highly intercorrelated. Logistic regression analysis was performed using LVH as the dependent variable to analyze the association between albumin-adjusted serum calcium level and LVH after adjusting for additional variables such as Alb/Cr, creatinine, albumin, SBP, age, gender, smoking, the use of ACEI/ARB medication, dyslipidemia, HbA1c, obesity and HOMA-IR. Odds ratios (OR) with 95% confidence intervals (CI) were calculated for the relative risk of elevated albumin-adjusted serum calcium level with LVH. The abilities to predict LVH of albumin-adjusted serum calcium, Alb/Cr and SBP were evaluated using the area under the curve (AUC) in the receiver operating characteristic (ROC) curve. Data were processed using Statistical Product and Service Solutions (SPSS) version 19.0. All tests were two-sided, P < 0.05 was considered statistically significant.

## Results

### Sample characteristics

The subjects in this study were 486 men and 347 women with T2DM (mean ages, 68.2 ± 13.9 and 67.0 ± 10.5, respectively; Table 1). The duration of diabetes was 10.4 ± 8.5 years. Hypertension, micro-albuminuria, dyslipidemia and obesity were present in 429 (51.5%), 476 (57.1%), 448 (53.8%) and 124 (14.9%) patients, respectively.

**Table 1:** **Baseline characteristics of subjects categorized by LVH and LV geometry**

**Characteristics**		**LVH**			**LV geometry**				
		**No**	**Yes**	**P value**	**Normal**	**Concentric remodeling**	**Eccentric hypertrophy**	**Concentric hypertrophy**	**P value**
Age (year)		67.8 ± 13.6	67.6 ± 10.8	0.823	67.6 ± 13.4	68.3 ± 14.0	66.5 ± 10.4	68.5 ± 11.0	0.188
Male, n (%)		371 (71.3)	115 (36.7)	<0.001	243 (71.7)	128 (70.7)	47 (33.6)	68 (39.3)	<0.001
Hypertension, n (%)		237 (45.6)	192 (61.3)	<0.001	151 (44.5)	86 (47.5)	79 (56.4)	113 (65.3)	<0.001
ACEI/ARB use, n (%)		219 (42.1)	159 (50.8)	0.015	139 (41.0)	80 (44.2)	59 (42.1)	100 (57.8)	0.003
Smoking, n (%)		116 (22.3)	74 (23.6)	0.657	72 (21.2)	44 (24.3)	36 (25.7)	38 (22.0)	0.695
Duration of diabetes (year)		9.8 ± 8.1	11.4 ± 9.0	0.010	9.4 ± 7.8	10.6 ± 8.7	11.5 ± 9.4	11.3 ± 8.7	0.023
SBP (mmHg)		134.7 ± 19.5	140.7 ± 19.3	<0.001	134.3 ± 19.4	135.4 ± 19.6	141.4 ± 21.4	140.2 ± 17.5	<0.001
DBP (mmHg)		78.7 ± 10.9	80.3 ± 11.2	0.048	79.2 ± 10.4	77.7 ± 11.7	78.6 ± 11.2	81.6 ± 11.0	0.008
BMI (Kg/M^2^)		24.2 ± 3.3	24.7 ± 4.5	0.079	24.0 ± 3.0	24.4 ± 3.8	25.2 ± 4.1	24.3 ± 4.8	0.038
Obesity, n (%)		56 (10.8)	68 (21.7)	<0.001	33 (9.7)	23 (12.7)	38 (27.1)	30 (17.3)	<0.001
Laboratory									
	FPG (mmol/L)	7.85 ± 3.11	7.78 ± 3.21	0.785	7.97 ± 3.31	7.63 ± 2.67	7.37 ± 2.43	8.12 ± 3.70	0.369
	lg HOMA-IR	0.56 ± 0.32	0.61 ± 0.34	0.069	0.56 ± 0.33	0.57 ± 0.30	0.58 ± 0.31	0.62 ± 0.36	0.166
	HbA1c (%)	8.16 ± 1.89	8.08 ± 1.98	0.518	8.17 ± 1.95	8.16 ± 1.78	8.03 ± 1.97	8.12 ± 2.00	0.898
	Albumin (g/L)	39.19 ± 4.70	37.66 ± 5.24	<0.001	39.31 ± 4.62	38.96 ± 4.86	37.14 ± 5.46	38.08 ± 5.03	<0.001
	Uric acid (μmol/L)	335.63 ± 100.71	342.15 ± 104.57	0.373	332.59 ± 101.36	341.31 ± 99.52	345.48 ± 110.94	339.45 ± 99.37	0.589
	Creatinine (μmol/L)	81.23 ± 22.28	84.10 ± 27.90	0.123	80.42 ± 21.41	82.77 ± 23.80	86.91 ± 30.03	81.82 ± 25.91	0.531
	lg Alb/Cr (mg/g)	1.57 ± 0.70	1.94 ± 0.63	<0.001	1.49 ± 0.68	1.72 ± 0.73	1.99 ± 0.62	1.90 ± 0.63	<0.001
	Micro-albuminuria, n (%)	246 (47.3)	230 (73.5)	< 0.001	147 (43.4)	99 (54.7)	103 (73.6)	127 (73.4)	< 0.001
	TG (mmol/L)	1.76 ± 1.11	1.95 ± 1.30	0.026	1.79 ± 1.21	1.71 ± 0.89	1.90 ± 1.43	1.99 ± 1.18	0.113
	TC (mmol/L)	4.75 ± 1.18	4.91 ± 1.13	0.065	4.69 ± 1.19	4.87 ± 1.15	4.96 ± 1.15	4.87 ± 1.12	0.093
	HDL-C (mmol/L)	1.11 ± 0.30	1.11 ± 0.46	0.781	1.11 ± 0.29	1.12 ± 0.31	1.14 ± 0.59	1.08 ± 0.32	0.301
	LDL-C (mmol/L)	2.70 ± 0.86	2.77 ± 0.83	0.221	2.67 ± 0.87	2.76 ± 0.83	2.80 ± 0.79	2.75 ± 0.86	0.35
	Dyslipidemia, n (%)	264 (50.8)	184 (58.8)	0.025	171 (50.4)	93 (51.4)	77 (55.0)	107 (61.8)	0.088
	albumin-adjusted-Calcium (mg/dL)	8.93 ± 0.35	9.09 ± 0.40	< 0.001	8.92 ± 0.36	8.96 ± 0.32	9.04 ± 0.41	9.13 ± 0.39	< 0.001
	Phosphate (mg/dL)	3.54 ± 0.47	3.57 ± 0.44	0.420	3.52 ± 0.46	3.58 ± 0.48	3.58 ± 0.43	3.56 ± 0.45	0.503
	Calcium-Phosphate product (mg/dL)	31.69 ± 4.64	32.44 ± 4.16	0.016	31.46 ± 4.55	32.11 ± 4.79	32.33 ± 4.07	32.52 ± 4.23	0.007

Significant difference in albumin-adjusted serum calcium was observed between non-LVH and LVH group (8.93 ± 0.35 *vs.* 9.09 ± 0.40 mg/dl, p < 0.001, Table 1). Percentage of the subjects with hypertension, micro-albuminuria, dyslipidemia, obesity and the use of ACEI/ARB were higher in subjects with LVH (eccentric or concentric hypertrophy) than those without LVH (normal or concentric remodeling). The patients with LVH also had higher levels of SBP, DBP, Alb/Cr, TG, and serum calcium-phosphate product, longer duration of diabetes, and lower levels of serum albumin.

Compared to subjects in albumin-adjusted serum calcium quartile 1 (8.42 – 8.69 mg/dl), those in quartile 4 (9.23 – 10.42 mg/dl) had significant higher percentage of LVH (53.5% *vs.* 23.0%, p < 0.001, Table 2). FPG, HOMA-IR, albumin, Alb/Cr, TC, LDL-C, phosphate, percentage of the subjects with LVH and micro-albuminuria, and duration of diabetes differed across albumin-adjusted serum calcium quartiles. From quartile 1 to quartile 4, percentage of the subjects with LVH and micro-albuminuria, levels of Alb/Cr, TC, LDL-C, FPG, HOMA-IR and phosphate have significant overall upward tendencies; moreover, levels of serum uric acid, creatinine and TG, and percentage of the subjects with dyslipidemia also have overall upward tendencies but non-significant.

**Table 2:** **Characteristics of subjects categorized by albumin-adjusted serum calcium quartiles**

**Characteristics**			**Albumin-adjusted calcium concentration (mg/dL)**						**P value**
			**8.42-8.69**		**8.70-8.93**		**8.94-9.22**	**9.23-10.42**	
**n**			**213**		**237**		**181**	**202**	
Age (year)			67.9 ± 11.4		66.6 ± 13.3		68.3 ± 12.6	68.4 ± 12.9	0.188
Male, n (%)			142 (66.7)		143 (60.3)		99 (54.7)	102 (50.5)	0.006
Hypertension, n (%)			105 (49.3)		120 (50.6)		89 (49.2)	115 (56.9)	0.353
ACEI/ARB use, n (%)			101 (47.4)		98 (41.4)		76 (42.0)	103 (51.0)	0.150
Smoking, n (%)			57 (26.8)		54 (22.8)		37 (20.4)	42 (20.8)	0.402
Duration of diabetes (year)			10.1 ± 8.0		10.4 ± 9.1		10.7 ± 8.1	10.4 ± 8.7	0.041
SBP (mmHg)			137.1 ± 19.2		137.8 ± 19.6		135.4 ± 21.6	137.2 ± 18.3	0.672
DBP (mmHg)			79.2 ± 10.0		79.6 ± 11.2		79.5 ± 12.2	78.8 ± 10.8	0.894
BMI (kg/m^2^)			24.5 ± 3.4		24.5 ± 4.1		24.3 ± 4.0	24.0 ± 3.8	0.105
Obesity, n (%)			25 (11.7)		37 (15.6)		31 (17.1)	31 (15.3)	0.472
Laboratory									
		FPG (mmol/L)	7.04 ± 1.97		7.57 ± 2.82		7.88 ± 3.04	8.92 ± 4.16	< 0.001
		lg HOMA-IR	0.53 ± 0.30		0.57 ± 0.32		0.61 ± 0.33	0.63 ± 0.36	0.011
		HbA1c (%)	8.12 ± 1.96		7.94 ± 1.74		8.17 ± 1.95	8.34 ± 2.06	0.181
		Albumin (g/L)	39.72 ± 4.42		38.90 ± 5.44		38.91 ± 4.16	36.85 ± 5.14	0.002
		Uric acid (μmol/L)	320.68 ± 95.21		336.17 ± 97.88		336.90 ± 98.26	359.72 ± 113.81	0.913
		Creatinine (μmol/L)	78.36 ± 20.31		83.13 ± 25.94		82.61 ± 24.86	85.24 ± 26.32	0.531
		lg Alb/Cr (mg/g)	1.60 ± 0.71		1.65 ± 0.72		1.79 ± 0.68	1.83 ± 0.66	< 0.001
		Micro-albuminuria, n (%)	107 (50.2)		126 (53.2)		113 (62.4)	130 (64.4)	0.007
		TG (mmol/L)	1.79 ± 1.10		1.85 ± 1.17		1.73 ± 1.05	1.94 ± 1.39	0.329
		TC (mmol/L)	4.59 ± 1.12		4.89 ± 1.07		4.77 ± 1.26	4.98 ± 1.19	0.003
		HDL-C (mmol/L)	1.08 ± 0.31		1.12 ± 0.28		1.19 ± 0.56	1.06 ± 0.27	0.301
		LDL-C (mmol/L)	2.63 ± 0.82		2.77 ± 0.79		2.62 ± 0.92	2.88 ± 0.85	0.004
		Dyslipidemia, n (%)	114 (53.5)		123 (51.9)		87 (48.1)	124 (61.4)	0.060
		albumin-adjusted-Calcium (mg/dL)	8.57 ± 0.08		8.84 ± 0.07		9.10 ± 0.08	9.52 ± 0.26	< 0.001
		Phosphate (mg/dL)	3.48 ± 0.43		3.54 ± 0.44		3.61 ± 0.47	3.59 ± 0.49	0.021
LV dimension									
	LVIDD (mm)		47.56 ± 4.19	47.03 ± 4.82		47.22 ± 4.57		47.99 ± 4.42	0.142
	LVIDS (mm)		29.89 ± 4.20	30.09 ± 3.62		30.49 ± 4.32		30.89 ± 4.46	0.066
	IVSD (mm)		10.27 ± 1.45	10.84 ± 1.52		10.66 ± 1.59		11.13 ± 1.73	< 0.001
	PWTD (mm)		9.48 ± 1.24	9.91 ± 1.34		9.93 ± 1.22		10.09 ± 1.45	< 0.001
	RWT		0.40 ± 0.06	0.43 ± 0.08		0.42 ± 0.06		0.42 ± 0.07	< 0.001
	LVMI (g/m^2^)		94.20 ± 19.02	101.51 ± 21.62		101.77 ± 22.65		111.21 ± 27.93	< 0.001
	LVEF (%)		65.77 ± 7.45	64.68 ± 6.97		63.69 ± 8.26		64.87 ± 7.53	0.056
LV geometry									
	Normal (%)		54.0	40.9		35.4		31.2	< 0.001
	Concentric remodeling (%)		23.0	21.5		27.6		15.3	
	Eccentric hypertrophy (%)		12.2	18.1		15.5		21.3	
	Concentric hypertrophy (%)		10.8	19.4		21.5		32.2	
LVH, (%)			23.0	37.6		37.0		53.5	< 0.001

### Serum calcium and metabolism-related parameters

Table 3 showed that albumin-adjusted serum calcium level was significantly and positively correlated with metabolism-related parameters such as FPG (r = 0.207, p < 0.001), HOMA-IR (r = 0.137, p < 0.001), uric acid (r = 0.165, p < 0.001), TG (r = 0.100, p = 0.004), TC (r = 0.119, p = 0.001) and LDL-C (r = 0.094, p = 0.007).

**Table 3:** **Correlation coefficients between albumin-adjusted serum calcium and various parameters**

	**Albumin-adjusted serum calcium**	
	**r**	**P value**
Age (year)	0.025	0.477
Duration of diabetes (year)	−0.004	0.900
SBP (mmHg)	0.022	0.522
DBP (mmHg)	−0.011	0.751
BMI (kg/m^2^)	−0.006	0.862
FPG (mmol/L)	0.207	< 0.001
lg HOMA-IR	0.137	< 0.001
HbA1c (%)	0.040	0.254
Uric acid (μmol/L)	0.165	< 0.001
TG (mmol/L)	0.100	0.004
TC (mmol/L)	0.119	0.001
HDL-C (mmol/L)	−0.043	0.212
LDL-C (mmol/L)	0.094	0.007

### LVMI and LVH

As we know, greater LVMI (a widely used method to assess LVH) corresponded to higher severity of LVH. From albumin-adjusted serum calcium quartile 1 to quartile 4, LVMI significantly (p < 0.001) increased from 94.20 ± 19.02 g/m^2^ to 111.21 ± 27.93 g/m^2^ (Table 2).

After the initial univariate linear regression analysis to select variables from factors including albumin-adjusted serum calcium, uric acid, the use of ACEI or ARB medication, age, gender, smoking, HbA1c, obesity, HOMA-IR, phosphate, creatinine, albumin, Alb/Cr, SBP, DBP, and dyslipidemia, the stepwise multiple linear regression (Table 4) showed that higher LVMI was associated with albumin-adjusted serum calcium, Alb/Cr, creatinine, obesity, serum albumin and dyslipidemia. There was no obvious collinearity among these predictors. Albumin-adjusted serum calcium was an independent factor that could influence LVMI, with a standardized regression coefficient at 0.193 (p < 0.001).

**Table 4:** **The stepwise multiple linear regression for LVMI**

**Variable**	**Unstandardized coefficients**		**Standardized coefficients**	***t*** **value**	***p*** **value**	**95.0% CI for B**	**Collinearity statistics**	
	**B**	**Std. error**	**β**				**Tolerance**	**VIF**
lg Alb/Cr	7.430	1.145	0.219	6.486	< 0.001	5.182 - 9.678	0.877	1.140
Albumin- adjusted serum calcium	12.141	2.039	0.193	5.953	< 0.001	8.138 - 16.143	0.948	1.055
Creatinine	0.117	0.032	0.121	3.609	< 0.001	0.053 - 0.180	0.889	1.124
Obesity	5.130	2.109	0.077	2.433	0.015	0.991 - 9.269	0.994	1.007
Albumin	−0.397	0.165	−0.083	−2.408	0.016	−0.721 - −0.073	0.836	1.196
Dyslipidemia	3.329	1.536	0.070	2.168	0.030	0.315 - 6.343	0.955	1.047

VIF: variance inflation factor.

The logistic regression analysis (Table 5) shows the ORs (95% CI) for LVH according to changes in albumin-adjusted serum calcium levels when calcium is a categorical variable (quartiles) or a continuous variable (1 mg/dl). In contrast to subjects in quartile 1 (8.42-8.69 mg/dl), there were significantly increased risk of LVH with subjects in quartile 2 [(8.70-8.93 mg/dl), OR (95% CI) = 1.836 (1.152-2.924), p = 0.011] and quartile 4 [(9.23-10.42 mg/dl), OR (95% CI) = 2.909 (1.792-4.720), p < 0.001], after adjusted for possible confounding factors including Alb/Cr, creatinine, albumin and SBP in model 1, further adjusted for age, gender and smoking and the use of ACEI or ARB medication in model 2, and furthermore adjusted for dyslipidemia, HbA1c, obesity and HOMA-IR in model 3. Subjects in quartile 3 (8.94-9.22 mg/dl) also had a tendency (OR = 1.624) to develop LVH compared to those in quartile 1, but the P value was non-significant (p = 0.054). When calcium was analyzed as a continuous variable, in model 3, for every 1 mg/dl increase in the albumin-adjusted serum calcium level, the OR (95% CI) for LVH was [2.400 (1.552-3.713), p < 0.001]. Of note, this association between calcium and LVH maintained significantly in model 1, model 2 and model 3, whether the albumin-adjusted serum calcium was analyzed as a quartile-categorized variable or a continuous variable.

**Table 5:** **OR (95% CI) of LVH according to albumin-adjusted serum calcium concentration**

**Model**		**Quartiles of albumin-adjusted serum calcium (mg/dL)**			**Continuous variable**
	**8.42-8.69**	**8.70-8.93**	**8.94-9.22**	**9.23-10.42**	
n	213	237	181	202	833
Crude model	1.000 (reference)	2.013 (1.331-3.043), 0.001	1.967 (1.268-3.052), 0.003	3.845 (2.521-5.865), < 0.001	3.063 (2.086-4.500), < 0.001
Model 1	1.000 (reference)	2.018 (1.313-3.102), 0.001	1.848 (1.171-2.915), 0.008	3.483 (2.237-5.425), < 0.001	2.727 (1.824-4.077), < 0.001
Model 2	1.000 (reference)	1.864 (1.182-2.942), 0.007	1.590 (0.979-2.582), 0.061	2.903 (1.806-4.665), < 0.001	2.449 (1.597-3.756), < 0.001
Model 3	1.000 (reference)	1.836 (1.152-2.924), 0.011	1.624 (0.992-2.658), 0.054	2.909 (1.792-4.720), < 0.001	2.400 (1.552-3.713), < 0.001

Values are OR (95% CI) and P value.

Model 1: adjusted for lg Alb/Cr, creatinine, albumin, and SBP.

Model 2: further adjusted for age, gender and smoking and the use of ACEI/ARB medication.

Model 3: further adjusted for Dyslipidemia, HbA1c, Obesity and lg HOMA-IR.

**Figure 1:**
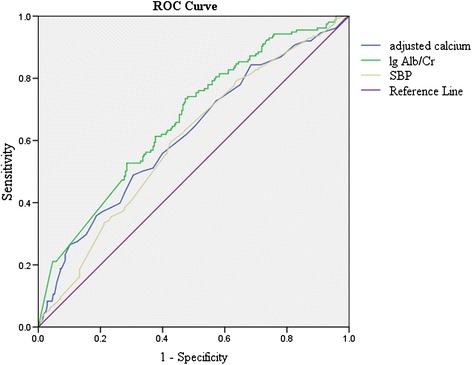
ROC curves of the ability of albumin-adjusted serum calcium, Alb/Cr and SBP to predict LVH.

To evaluate the predictive performance of albumin-adjusted serum calcium for LVH, the AUC in ROC curve was calculated. Alb/Cr and SBP which representing albuminuria and hypertension respectively and being well-known risk factors for LVH were used as references (Figure 1). The AUCs of albumin-adjusted serum calcium, Alb/Cr and SBP were as follows: 0.617 [95% CI (0.577-0.656), p < 0.001]; 0.668 [95% CI (0.630-0.705), p < 0.001]; 0.597 [95% CI (0.558-0.636), p < 0.001]. Approximate to Alb/Cr and SBP, albumin-adjusted serum calcium significantly predicted LVH.

### LV Geometry

From normal LV geometry to concentric remodeling, eccentric hypertrophy and concentric hypertrophy, albumin-adjusted serum calcium levels significantly (p < 0.001) increased from 8.92 ± 0.36 to 8.96 ± 0.32, 9.04 ± 0.41 and 9.13 ± 0.39 mg/dl, respectively (Table 1). Phosphate concentrations had no significant differences among groups categorized by LV geometry.

Table 2 summarized echocardiographic parameters categorized by albumin-adjusted serum calcium quartiles. IVSD, PWTD, RWT, and LVMI of subjects in quartile 4 (9.23-10.42 mg/dl) were significantly higher than those in quartile 1 (8.42-8.69 mg/dl). As an important indicator of systolic function, LVEF had a non-significant (p = 0.056) overall downward tendency from quartile 1 to quartile 4, but was significant different between quartile 1 and quartile 3 (65.77 ± 7.45 *vs.* 63.69 ± 8.26%, p = 0.009). LVIDS had a non-significant (p = 0.066) overall upward tendency across albumin-adjusted serum calcium quartiles, but was significant different between quartile 1 and quartile 4 (29.89 ± 4.20 *vs.* 30.89 ± 4.46 mm, p = 0.019).

From albumin-adjusted serum calcium quartile 1 to quartile 4, percentage of the subjects with normal LV geometry decreased significantly from 54.0% to 31.2%; by contrast, percentage of the subjects with LV eccentric hypertrophy and concentric hypertrophy increased sharply from 12.2% to 21.3%, 10.8% to 32.2%, respectively (Table 2).

## Discussion

LVH is very common in T2DM [2-6]. Very recent studies demonstrate that an increase in serum calcium concentrations is associated with an increased risk of T2DM [11,12]. Hence an important question arises whether elevated serum calcium contributes to LVH prevalence in T2DM.

To the best of our knowledge, this is the first analysis of the association between changes in albumin-adjusted serum calcium levels and the risk of LVH that focused specifically on T2DM patients with normocalcemia and normophosphatemia. Our results showed a clear association between the elevated albumin-adjusted serum calcium levels and the increased risk of LVH. Such an association is independent of the effect of Alb/Cr, SBP, dyslipidemia, the use of ACEI/ARB medication, age, gender, smoking, HbA1c, obesity and HOMA-IR.

In our study, patients with LVH had significantly higher levels of albumin-adjusted serum calcium (as well as calcium-phosphate product), and higher but non-significant levels of serum phosphate than those without LVH. On the other hand, patients in the highest serum calcium quartile had significantly higher percentage of LVH than those in the lowest quartile. Previous studies have demonstrated a high incidence of LVH both in patients with hypercalcemia (often hypophosphatemia) caused by primary hyperparathyroidism and in patients with hyperphosphatemia (often hypocalcemia) caused by uremia and secondary hyperparathyroidism [21-25]. Though parathyroid hormone per se plays an important role, an acute impairment in left ventricular diastolic function may be predominantly due to elevated serum calcium level but not plasma parathyroid hormone [26,27]. Moreover, hyperphosphatemia is an independent risk factor for LVH [23,28], and increased calcium-phosphate product contributes to LVH and left ventricular diastolic function impairment [23,24]. However, in subjects with normal ranges of serum calcium and phosphate, the relationship between serum calcium/phosphate and LVH is unknown. Our study indicates that elevated serum calcium though in normal range is related to LVH prevalence in T2DM.

LVH is an ominous predictor of cardiovascular outcomes and is a multifactorial process with many causes. However, it has often been ignored, to some extent, because of two common misconceptions that LVH only occurs in severe hypertension and ACEI/ARB can cure LVH [3]. Although hypertension is regarded as a leading cause of LVH [1], evidence demonstrates that blood pressure explains only 25% of the variability in LV mass [29].

In the current study, the stepwise multiple linear regression analysis showed a significantly association between albumin-adjusted serum calcium and LVMI. Moreover, consistent with previous studies [1,30-34], renal insufficiency (characterized by higher Alb/Cr and creatinine as well as lower serum albumin), obesity, and dyslipidemia were also related to an increasing of LVMI. Given that 57.1% subjects (47.3% in non-LVH, 73.5% in LVH) in this study had micro-albuminuria, it was unsurprising that Alb/Cr had a highest standardized coefficient among all independent variables in the stepwise multiple linear regression. As a major LVH risk, elevated albumin-adjusted serum calcium is second only to albuminuria (standardized coefficients: 0.193 and 0.219, respectively). Albumin-adjusted serum calcium, hypertension (indexed by SBP) and albuminuria (indexed by Alb/Cr) had similar predictive performance for LVH which evaluated by AUCs in ROC curve (0.617, 0.597 and 0.668, respectively). In the Insulin Resistance Atherosclerosis Study, individuals with albumin-adjusted serum calcium level ≥ 9.5 mg/dl were at significantly increased risk (OR = 1.79) of developing diabetes [12]. In our study, subjects with calcium level (9.23-10.42 mg/dl) had a sharply increased risk (OR = 2.909) for LVH than subjects with calcium level (8.42-8.69 mg/dl); and for per 1 mg/dl increase in calcium level, the OR for LVH was 2.400. These results suggested that moderate changes of calcium level, even in the normal range, had important clinical significance.

In this study, women had a higher prevalence of LVH than men (57.1% *vs.* 23.7%, p < 0.001). This observed gender differences could be partially accounted for by the greater prevalence of micro-albuminuria (63.1% *vs.* 52.9%, p = 0.003) and obesity (19.0% *vs.* 11.9%, p = 0.005), as well as the poorer glucose control (HbA1c: 8.31 ± 1.90 *vs.* 8.00 ± 1.93%, p = 0.021) in women. These results are consistent with two previous large-scale hospital-based studies, which showed that women with T2DM were less likely than men to achieve therapeutic targets and diabetes conferred a greater increase of cardiovascular risk in women than in men [35,36]. Moreover, many studies supported a more pronounced impact of diabetes (as well as other metabolic syndrome factors) on left ventricular structure/function in women than in men [37-40]. However, it is interesting but unclear whether alterations in calcium homeostasis, like metabolic disorders, have a sex-specific effect on left ventricular hypertrophy. Thus, our future study will explore in detail the interaction of these factors on development of LVH.

As an essential characteristic of T2DM, insulin resistance is accompanied with an increase in intracellular calcium [41,42]. Ours and others [10,43] studies demonstrated a positive correlation between insulin resistance (indexed by HOMA-IR) and serum calcium level. In consistent with this, serum calcium level is inversely associated with insulin sensitivity measured with euglycaemic-hyperinsulinaemic clamp in the normocalcemic general population [44]. However, it is controversial whether insulin resistance plays a role in the pathogenesis of LVH, with some studies [45,46] showing a clear relationship while others [5,47] having no significant association between insulin resistance and LVH. In our study, there was an increased [OR (95% CI) = 1.486 (0.969-2.280)] but non-significant (p = 0.069) risk for LVH in individuals with insulin resistance (logarithmically transformed HOMA-IR as a continuous variable). Further studies are warranted to validate the association between insulin resistance and LVH.

The calcium ion is an essential regulator for a great diversity of physiology processes, including contraction of cardiac, skeletal, and smooth muscle; neurotransmitter release; various forms of endocrine and exocrine secretion; and automaticity of nerve and muscle [48]. We can speculate about the mechanisms underlying the association between serum calcium and LVH. On the one hand, the alteration in serum calcium could act as a common link among various metabolic disorders. In our study, albumin-adjusted serum calcium level was significantly and positively correlated with metabolism-related parameters such as FPG, HOMA-IR, uric acid, TG, TC and LDL-C. These results are in line with previous studies that show a direct association between albumin-adjusted serum calcium levels and metabolism parameters of blood lipids and glucose [8,10,43,49,50]. A health screening survey with over 18,000 adult participants showed that serum calcium concentration was positively related to serum glucose and cholesterol concentrations [8]. In another study with 1,182 healthy subjects, after adjusting for 25-OH vitamin D and parathyroid hormone, significant positive correlations between glucose and insulin resistance (indexed by HOMA-IR) with calcium were found in both sexes, whereas an inverse correlation between beta-cell function (indexed by HOMA-β) and calcium was found only in women [43]. Conversely, only in men, serum calcium level was positively associated with impaired glucose metabolism (indexed by fasting blood glucose) in a cross-sectional study with 480 T2DM patients; and this association was independent of PTH or bone metabolism [10]. In the national prevention program of diabetes in Finland with 2896 participants, serum calcium level is even associated with metabolic syndrome and its components except HDL-C, after adjustment for age, physical activity, alcohol, vitamin D intake, calcium intake, and smoking [49]. Given that various metabolic abnormalities, such as diabetes, central obesity, dyslipidemia and hyperuricemia have been reported in association with LVH, even in the absence of hypertension [1,3,32,51,52], these results suggest that increased serum calcium level may correlate with LVH through metabolic abnormalities. On the other hand, left ventricular function is sensitive to disorders in calcium metabolism [27]. The contraction and relaxation of both vascular smooth muscle cells and cardiomyocytes are highly dependent on cytosolic calcium homeostasis [26,28]. Calcium has a hypertrophic action on heart cells [21]. A positive calcium balance can accelerate soft-tissue and vascular calcification which may potentially lead to impaired left ventricular relaxation even without hypercalcemia [28,53]. Calcium increase is a potential trigger of the translocation of pro-hypertrophic transcription factors which involved in the cardiac development to the nucleus [54]. Sustained elevation in intracellular calcium may lead to excessive activation of calcineurin pathway in the heart, and cardiomyocytes from calcineurin transgenic hearts are disorganized and markedly hypertrophic [55,56]. Calcium homeostasis impairment also is a prominent feature in the transition from compensatory hypertrophy to heart failure [54]. In additional, hemodynamics can be altered by variations in serum calcium through changes in left ventricular stroke volume and output [28]; and essential hypertension can alter calcium metabolism which in turn provokes the development of myocardial hypertrophy [57].

Our study chose a selected population (T2DM patients with normal serum calcium, phosphate and creatinine, without macro-albuminuria). Without overt biochemical abnormalities or symptoms in these patients, alterations in calcium homeostasis are easy to be ignored in our clinical practice. These findings suggest that in T2DM patients equal attention should be paid to increased serum calcium and traditional risk factors for LVH such as albuminuria and hypertension.

Several limitations of this study must be taken into account when interpreting the results. First, in our study no measurements of serum levels of parathyroid hormone and Vitamin D are available for most of the population, so it is impossible to absolutely exclude confounding factors such as primary hyperparathyroidism and secondary hyperparathyroidism due to vitamin D deficiency. A previous study found 30 cases (0.6%) with normal serum calcium and inappropriately high serum PTH (normocalcemic primary hyperparathyroidism) in 5202 subjects, using the diagnostic criteria including serum calcium 2.50-2.60 mmol/l with PTH ≥ 35 ng/L and calcium < 2.50 mmol/l with PTH > 55 ng/L [58]. However, even without associated disturbances in serum PTH, serum calcium per se in the reference range is positively associated with cardiovascular endpoints such as myocardial infarction [59,60]. Since serum calcium levels are well-known lower or low-normal in individuals with secondary hyperparathyroidism caused by vitamin D deficiency and/or renal insufficiency, in our study, secondary hyperparathyroidism (as well as corresponding vitamin D deficiency and/or renal insufficiency) cannot account for the higher rate of LVH among patients in the higher serum calcium quartiles. Because the levels of serum calcium as well as phosphate are regulated in a coordinated way and there are important interactions between calcium and phosphate [61], to minimize the possibility that some abnormal conditions may influence the results, we excluded individuals with serum calcium and phosphate levels outside the normal range. Second, ionized calcium which is the physiologically active form [62], was not measured in our study. However, total serum calcium level (adjusted with serum albumin to which calcium is bound) is highly correlated with ionized calcium level, and is still widely used in current clinical practice. In several recent studies [10-12], albumin-adjusted total calcium is used for assess the association between serum calcium level and risk of T2DM. Third, the subjects in this study were Chinese, and our findings may not be relevant to other ethnic populations. Finally, the sample size in our study is moderate; the hospital-based study is susceptible to sample selection bias; and cause-effect relationship cannot be drawn from the cross-sectional study.

## Conclusions

In summary, our results extend previous identified association of calcium homeostasis with LVH from hyperparathyroidism with dyscalcemia to T2DM with normocalcemia. The increased albumin-adjusted serum calcium level, even within the physiological ranges, is independently associated with LVH prevalence in patients with T2DM.

## Abbreviation

LVH: Left ventricular hypertrophy
T2DM: Type 2 diabetes mellitus
LVM: Left ventricular mass
LVMI: Left ventricular mass index
LVIDD: Left ventricular internal diameter, diastolic
PWTD: Posterior wall thickness, diastolic
IVSD: Inter ventricular septum, diastolic
RWT: Relative wall thickness
AUC: Area under the curve
ROC: Receiver operating characteristic
OR: Odds ratio
CI: Confidence intervals
HOMA-IR: Homeostasis model assessment insulin resistance
HOMA-β: Homeostasis model assessment beta-cell function
PTH: Parathyroid hormone
Alb/Cr: Urine albumin per gram urine creatinine
TC: Total cholesterol
TG: Triglycerides
LDL-C: Low-density lipoprotein cholesterol
HDL-C: High-density lipoprotein cholesterol
FPG: Fasting plasma glucose
BSA: Body surface area
SD: Standard deviation
ACEI/ARB: Angiotensin converting enzyme inhibitor/ angiotensin receptor blocker
SBP: Systolic blood pressure
DBP: Diastolic blood pressure
